# Processing DNA Storage through Programmable Assembly in a Droplet‐Based Fluidics System

**DOI:** 10.1002/advs.202303197

**Published:** 2023-09-26

**Authors:** Minsang Yu, Doyeon Lim, Jungwoo Kim, Youngjun Song

**Affiliations:** ^1^ Standard Bioelectronics. Co. 511 Michuhol Tower, Gaetbeol‐ro 12 Incheon 21999 South Korea; ^2^ Department of Nano‐Bioengineering Incheon National University Academy‐ro 119 Incheon 22012 South Korea

**Keywords:** address primer scanning, DNA data storage, droplet‐controlled fluidics system, processing of DNA storage, splint ligation

## Abstract

DNA can be used to store digital data, and synthetic short‐sequence DNA pools are developed to store high quantities of digital data. However, synthetic DNA data cannot be actively processed in DNA pools. An active DNA data editing process is developed using splint ligation in a droplet‐controlled fluidics (DCF) system. DNA fragments of discrete sizes (100–500 bps) are synthesized for droplet assembly, and programmed sequence information exchange occurred. The encoded DNA sequences are processed in series and parallel to synthesize the determined DNA pools, enabling random access using polymerase chain reaction amplification. The sequencing results of the assembled DNA data pools can be orderly aligned for decoding and have high fidelity through address primer scanning. Furthermore, eight 90 bps DNA pools with pixel information (png: 0.27–0.28 kB), encoded by codons, are synthesized to create eight 270 bps DNA pools with an animation movie chip file (mp4: 12 kB) in the DCF system.

## Graphical abstract

DNA has gained interest as a high‐capacity data storage medium. DNA containing digital data can be assembled and ligated using a droplet‐controlled fluidics system, resulting in new DNA that contains the encoded information. The DNA pools encoding pixel images in the droplets are edited to create a DNA pool for pixel animation.

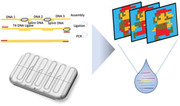

## 1 Introduction

Digital DNA data storage can store sequence information at the molecular level and has been a recent focus of interest because of its capacity for storing extremely dense information^[^ ^1^, ^2^ ^]^ and unique computing ability.^[^ ^3^ ^]^ Digital data is encoded into DNA as a packet of information, represented as size (structure)^[^ ^4^, ^5^, ^6^ ^]^ or sequence‐based DNA, and contains a combination of binary digits, similar to electronic data.^[^ ^4^, ^7^, ^8^ ^]^ Furthermore, to solve mathematical problems in Boolean logic operation and cell computing, single‐strand DNA (ssDNA) sequences, which can undergo hybridization and displacement reactions, have been developed as nanocomputing materials for diverse computational research, such as in neural networks.^[^ ^3^, ^9^, ^10^, ^11^, ^12^ ^]^ Despite the functional duality of DNA for computing and memory storage using sophisticated biological technology, processing digital data stored in DNA is not well studied. Most studies focused on data capacity,^[^ ^13^ ^]^ storage applications,^[^ ^14^ ^]^ and sequence encoding methods.^[^ ^1^ ^]^ For DNA editing, a DNA nanoswitch system has been developed with fluorescent monitoring or size (structure)‐based detection, which can be used as computing and storage system.^[^ ^15^ ^]^ These systems can process stored DNA data but have limited data capacity due to display limitations, such as electrophoresis lanes and many fluorescent probes. For high‐capacity storage, synthetic oligos are encoded with two bits of binary information in each sequence. For parallel storage, the synthetic oligo sequences are tethered with index sequences.^[^ ^16^ ^]^ Common forward and reverse primers are allocated to the synthetic oligo pool to retrieve the stored DNA data using biological methods such as polymerase chain reaction (PCR), a tool that exponentially amplifies the original DNA molecule. Additionally, next‐generation sequencing (NGS) is widely used to detect digital data stored in DNA.^[^ ^17^, ^18^, ^19^ ^]^ Furthermore, diverse encoding methods have been developed to reduce sequencing errors, such as homopolymers and high GC contents, to correct NGS sequencing results, and to ensure high accuracy.^[^ ^20^, ^21^ ^]^

Primer sequences have limitations in reducing DNA data density; however, they help improve data by enabling precise and targeted amplification and ensuring efficient stored information retrieval. Similar to digital data signal amplification using electronic circuits, data molecules with standard forward and reverse primers are amplified using PCR. Moreover, using orthogonal primers, PCR can select a set of DNA data sequences from pools.^[^ ^22^, ^23^ ^]^ Independent primers can perform computational functions such as Boolean logic operations for file selection,^[^ ^24^ ^]^ and primers can also be used for data deletion using index marker DNA hybridization for data security.^[^ ^25^ ^]^ However, high‐capacity DNA data storage has not been actively developed for data processing, such as editing or ordering data. Most studies on DNA data storage have focused on cold data storage, where files cannot be frequently accessed.^[^ ^26^ ^]^ Primers and sophisticated biotechnological methods can demonstrate that digital DNA has a functional duality that can be used for computing and data storage. Similar to processing in memory (PIM), recently introduced in electronic device systems to combat data processing limitations, the DNA data molecules, which have data stored in primer sequences, can be used for computing. Specialized devices may be helpful for DNA molecules to handle and manipulate individual DNA sequences. An electric field‐assisted DNA microarray has been developed for hybridization and displacement for short ssDNA sequence processing, allowing random access for three‐bit storage and computing.^[^ ^27^ ^]^ However, its ability to handle high‐capacity DNA and discrete data molecules is limited. A droplet‐based DNA handling system could be helpful for discrete data molecules. Regarding developing DNA storage systems, an electrowetting device can produce a DNA data storage system with 127 sites that can manipulate DNA data into a single pool.^[^ ^28^ ^]^

**Figure 1:**
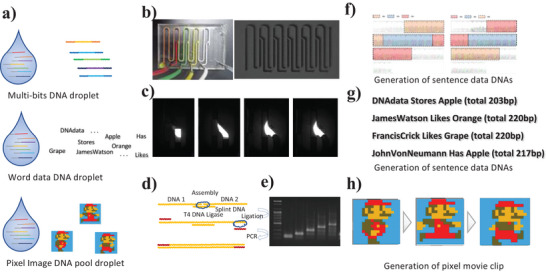
Programmable DNA data storage assembly in a droplet controlling fluidics (DCF) system. a) DNA data droplet of the diverse type format (top: size‐based bit data, center: sequence‐based encoded‐word data, bottom: sequence‐based encoded pixel art). b) The image and diagram of the DCF chip with multiple y‐junction channels. c) Images of droplet association in y‐junction. d,e) Diagram and gel image of DNA data assembly. f) Sanger sequencing results for the DNA bits programmable assembly. g,h) The NGS decoding results of the sentences DNA data (g), and the NGS decoding results of the DNA pool movie clip (h).

However, this system uses a limited electrode array to focus on the DNA data storage spot and droplet path. Thus, owing to the lack of DNA data editing technology, DNA data operating devices might be helpful to allow DNA data editing, such as DNA data connection. Here, we demonstrate a droplet device for processing DNA data (**Figure** **1**). The digitized droplets were associated with a fluidic system constructed using a 3D‐printed mold.^[^ ^11^ ^]^ The DNA data were ligated using split DNA ligation in the associated droplet. For size and structure‐based DNA storage, 100 bps ssDNAs were assembled with splint DNA in the fluidic chip, creating DNA molecules of varying lengths (100–500 bps), each able to display up to five bits of information. Furthermore, we demonstrate that the assembled DNA sequence order was altered using splint DNAs (Figure 1f). The encoded‐word DNA data were in series and parallel processed to produce diverse DNA sequences with sentence information to show the PIM storage for high‐density DNA data storage (Figure 1g). Finally, we demonstrate that the three DNA pools of the pixel image data, encoded by codon sequences, were created using animation DNA pools. Unlike word DNA data sequences, using an American Standard Code for Information Interchange (ASCII) code with error correction code (ECC), which applies 2^7^ Galois filed reed‐solomon (RS) code,^[^ ^29^ ^]^ the codon‐based sequence provided 1.33 bits per bp logical capacity to prevent homopolymers for repeated pixel information. Furthermore, splint address sequences are used for DNA assembly to process DNA data, scanning to search for flexible DNA payloads (unknown data sizes and positions), and aligning them to synchronize the sequences. Due to the capability of the programmable assembly of DNA, the droplet DNA ligation system can be used for DNA digital data storage. In addition, DNA data pool ligation can increase storage ability.^[^ ^30^ ^]^

## 2 Results

### 2.1 Digitized Droplet Controlling Fluidics System

**Figure 2:**
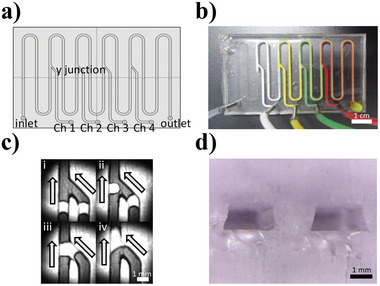
Digitized DCF system. a) Schematic diagram representing the four y‐junction channels in the DCF chip. b) Image of the DCF chip. c) Microscopy images of droplet association. d) Cross‐sectional image of the channels.

Digital data, a sequential combination of discrete “0” and “1” numbers, store information, including documents, pictures, and movie files. For example, letter information codes such as ASCII and Universal Transformation Format (UTF‐8)^[^ ^2^, ^3^, ^31^, ^32^, ^33^ ^]^ operate based on a set of sequentially digitized data. This data follows the bits string data format, including the most significant bit (MSB) and a lower significant bit (LSB). The droplet carrier and DNA molecules can be beneficial to store discrete data in DNA as discrete DNA information can be manipulated. A fluidics system operated by droplet carriers can be provided to ensure the programmable DNA droplet association to store DNA droplet data. We fabricated a droplet controlling polydimethylsiloxane (PDMS) fluidics system, casted using a 3D printed mold to control discrete DNA data molecules.^[^ ^15^, ^34^ ^]^ A detailed discussion regarding the process is provided in Experimental Section and Supporting Information 1. For 2 µl of DNA molecular droplets, a droplet controlling fluidics (DCF) channel was designed, which was 1 mm width and 1 mm height (**Figure** **2**a,b). For multiple DNA data droplet manipulation using the DCF chip, we designed a main channel with four y‐junctions, in which the droplet association can be operated. DNA droplets in the main DCF channel were ≈2 mm long with a 1 mm gap. Figure 2c represents the microscopy images of the droplet association at the DCF y‐junction. In DCF systems, DNA droplets storing discrete data can be used for programmable assembly of information through droplet association. The associations of the discrete DNA droplets can provide more intelligent DNA information systems, such as DNA computing^[^ ^35^ ^]^ and digital data storage^[^ ^36^ ^]^ using DNA hybridization and enzymatic reactions. DNA information storage through hybridization and enzymatic reactions in the DCF can serve as a multi‐bit DNA storage system^[^ ^37^, ^38^ ^]^ and as digital DNA molecular data storage.^[^ ^39^ ^]^

### 2.2 DNA Droplet Assembly using Splint Ligation

DNA ligation helps manipulate DNA molecular size and structure, and gel electrophoresis can display digitized DNA information, such as letter codes.^[^ ^15^, ^40^, ^41^ ^]^ In particular, splint DNA ligation can aid in programmable assembly using sequence information. Furthermore, DNA droplet carriers can be used as a discrete handling tool.^[^ ^31^, ^42^, ^43^ ^]^

**Figure 3:**
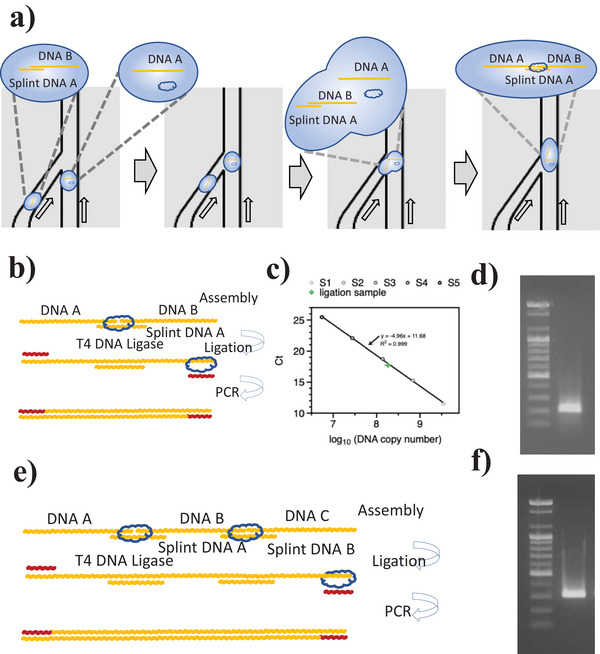
DNA droplet assembly using splint ligation. a) The DNA droplet in the DCF system. b) The splint ligation process with DNA A and B. c) Standard qPCR curve for the 200‐bps ligation sample (aliquoted to 1/40 000 for the droplet, n = 3). d) The gel electrophoresis results of the splint DNA‐ligated 200 bps fragment. e) The two series splint ligation experiments (DNA A, B, and C), and f) The gel electrophoresis results of the splint DNA‐ligated 300 bps fragment.

We demonstrated DNA droplet assembly with splint ligation in DCF for discrete programmable DNA information (**Figure** **3**). A single droplet with DNA A (100 bps ssDNA) and T4 DNA ligase in the main channel moved into the y‐junction. Another droplet with polynucleotide kinase‐treated DNA B (100 bps ssDNA), which included a pre‐assembled splint DNA sequence (30 bps ssDNA), was associated with the droplet in the main channel (Figure 3a). The two DNA sequences in the associated droplet were hybridized with splint DNA and ligated using T4 DNA ligase, which can be activated at room temperature^[^ ^32^ ^]^ (25 °C) for 20 min. The linked DNA molecules are thermostable and permanently connected through ligation (Figure 3b). The 200 bps DNA fragment ligation was confirmed and amplified using PCR. The copy number of the ligated 200 bp DNA fragment was quantified using quantitative PCR (qPCR) to measure the efficiency of splint ligation (Figure 3c). Due to using a high amount of T4 DNA ligase (400 units), the ligation efficiency was 18.5%. This result was calculated using a copy number of 1.67 ×10^12^, a decrease from the original copy number of 9.04 ×10^12^ (Figure S2, Supporting Information). Figure 3d shows the gel electrophoresis results of the ligated 200 bps DNA fragment. In the series process, three DNA fragments (A, B, and C) were ligated in the DCF system and verified through PCR. Figure 3f shows the gel electrophoresis results of the 300 bps DNA sequence, which has information tethered to DNA A, B, and C.

### 2.3 Multi‐Bit Droplets DNA Ligation

**Figure 4:**
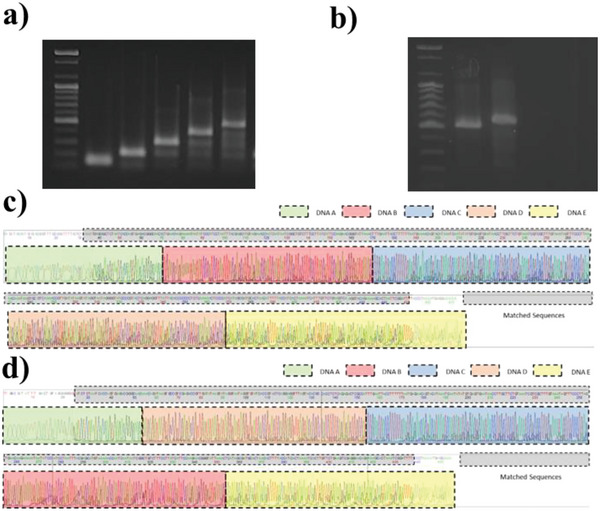
DNA ligation of multi‐bits droplets. a) 100–500 bps discrete DNA band results. b) Two different 500 bps DNA fragments after droplet DNA assembly. c,d) Sanger sequencing results for DNA fragments in the series order of DNA A, B, C, D, and E (c), and DNA fragments in the series order of DNA A, D, C, B, and E (d).

For multi‐bit DNA information, the discrete DNA bands in gel electrophoresis can conveniently display DNA information, such as alphanumeric characters.^[^ ^15^ ^]^ The gel electrophoresis results of five bits of DNA information displayed different DNA gel bands from 100 to 500 bps induced by programmable ligation (**Figure** **4**a). By combining ssDNA with splint DNA, which can be informationally tethered, stored digital DNA data can be displayed as binary data of 100 bps in length. Digital DNA data, such as alphanumeric characters encoded as the Baudot code,^[^ ^15^ ^]^ can be represented by the ligated DNA, which are 100 bps fragments incorporated into LSB and 500 bps into MSB.^[^ ^34^, ^35^, ^36^, ^42^ ^]^ The ligation is because the address sequence of DNA is assembled with another DNA fragment by the associated splint DNA, which contains the address information. For four serial ligations, the efficiency is expected to be at 0.1% without calculating the increasing ligation time for the DNA in the first droplet due to the low efficiency (18.5%) of a single splint ligation process. We have provided gel electrophoresis results with and without ligation and PCR to assess the feasibility of DNA association (Figure S3, Supporting Information). Lane 2 shows the assembled DNA bands with ligation, lane 3 represents the assembled DNA bands without ligation, lane 4 has a strong ligated DNA band, and lane 5 shows DNA bands of assembled DNA without ligation. The gel electrophoresis results show that PCR amplification can help assess whether the recovered ligated DNA fragments are high‐quality.

Moreover, due to the addressed splint DNA, the alignment order of the DNA sequence can be changed in DCF. Figure 4b shows the 500 bps DNA gel bands from different DNA ligation combinations. Figure 4c,d shows Sanger sequencing results for DNA fragments in the series order of DNA A, B, C, D, and E (lane 2 in Figure 4b) and DNA fragments in the series order of DNA A, D, C, B, and E (lane 3 in Figure 4b). Furthermore, the NGS read results were analyzed and displayed in Figures S4 and S5 (Supporting Information). The sequencing data corresponded to the original DNA information, maintaining its fidelity and accuracy. More detailed information is provided in Supporting Information 5. The splint DNA ligation method created serial information‐tethered DNA representing discrete DNA information to perform multi‐bit assembly. Furthermore, droplet assembly in the DCF system can provide sequence‐encoded DNA data storage.^[^ ^28^ ^]^

### 2.4 Programmable Assembly for DNA Word Data

**Figure 5:**
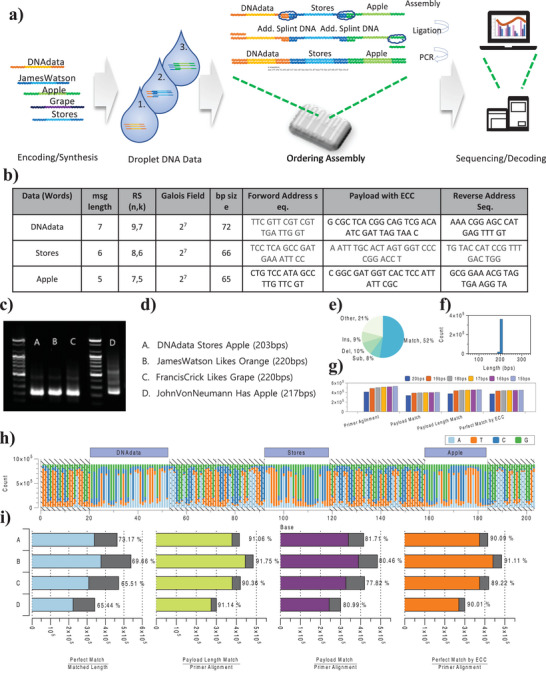
Programmable ordering assembly for sequence‐encoded word DNA data. a) The synthesis and decoding process of droplet assembly. b) The sequence and encoded information of DNA words. c) Gel electrophoresis results. d) The lengths of DNA sentence data fragments. e–g) Sequencing analysis of lane A. h,i) The consensus sequencing results of lane A h), and the sequencing analysis results (i).

The DNA word data sequences, encoded by ASCII,^[^ ^38^ ^]^ were designed in orthogonal address primer sequences to exhibit programmable word information combinations. For error data correction (one character or seven bits), the word data sequences were encoded with a seven‐bps ECC redundancy using the shortened RS code.^[^ ^29^ ^]^ The Galois field was defined as 2^7^ due to the symbol of the seven bits ASCII encoding method (0–127). Similar to previous multi‐bit droplet DNA ligation processes, the DNA word data in the droplet can be assembled with splint DNA in DCF (**Figure** **5**a). Figure 5b and Table S2 (Supporting Information) provide the word list and sequence information. The ECC code and the encoding/decoding method are presented in more detail in Supporting Information 7.

Figure 5c shows the gel electrophoresis results to produce various DNA sentence data. The 203 bps DNA sentence data (lane A) fragment, synthesized using two splint ligation processes in DCF, represented “DNAdata Stores Apple.” The synthesized diverse DNA sentence data fragments represented “JamesWatson Likes Orange,” shown in lane B (220 bps), “FrancisCrick Likes Grape,” shown in lane C (220 bps), and “JohnVonNeumann Has Apple,” shown in lane D (217 bps). The DNA sentence data fragments were analyzed through NGS. The resultant sequences, trimmed with forward and reverse address sequences, were decoded to digital data using consensus sequences (Figure 5d,h; Figure S7, Supporting Information). The data sizes were not defined (the sequence length of the DNA data can be modulated, depending on data size); therefore, we searched for the data sequence positions and sizes using the address sequence information to retrieve the data stored in DNA sequences. After the first 20 bps of the forward was scanned until 20th bases, the reverse address sequences were scanned from the 21st base to the end of the NGS sequences until the corresponding reverse address sequences were determined to identify the address sequences in NGS raw data. The protocol is detailed in “Alignment Using Address Sequences.” Figure 5e shows the analysis of the sequencing results for the DNA sentence data fragment in lane A. The degree of perfectly matched sequences was 52.4%, and the substitution error was 7.6%. One and two deletion errors were 10.2%, and the insertion error was 8.8% in the NGS raw data. Figure S6 (Supporting Information) provides the sequencing results of other DNA sentence data fragments. The most frequently read sequences were 203 bps (59.2%) in length (Figure 5f). Filters can be used to ensure highly reliable results, designed to match the exact address sequences (20 bps), albeit at the cost of reduced coverage. Figure 5g provides the increased number of counts for address sequence alignment, perfect matches, payload data length matches, and the results of applying ECC to the matched address sequence, which was 15–20 bps. Figures S7 and S9 (Supporting Information) show the consensus sequencing results, including payload data with and without address sequence alignment. A more detailed explanation and count results of other DNA sentence data are provided in Supporting Information 10.

The consensus sequencing result for the DNA sentence data “DNAdata Stores Apple” is provided in Figure 5h. Owing to the high decoding yield, including the high percentage of perfect match sequences, applying the ECC was unnecessary to recover the ACSII data. The sequencing results with ECC are analyzed in more detail in Figure 5i. The perfectly‐matched DNA sequences comprise 73.17%, 69.66%, 65.51%, and 65.44% of lanes A–B, respectively. The payload matching the length in the DNA sentence data, resorted by address alignment, comprises 91.06%, 91.75%, 90.36%, and 91.14% of lanes A–D, respectively. Furthermore, we provided the number of matched sequence data encoded with an ECC (Figure S10, Supporting Information). The percentages of match sequences with ECC applied were increased from 81.71% to 90.09% in lane A, 80.46%–91.11% in lane B, 77.82%–89.22% in lane C, and 80.99%–90.01% in lane D. We conducted DNA synthesis of various sentence structures, using the DNA words. Their information conveyed the intended meaning even if the sentences were not grammatically correct.

### 2.5 Synthesizing Multiple DNA Data Pools

**Figure 6:**
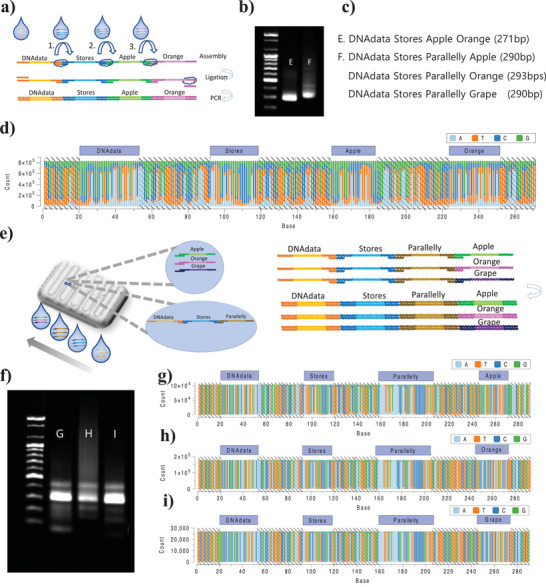
Serial and parallel DNA data storage. a) A diagram of the ligation of multiple serial word data DNA fragments. b) The gel electrophoresis results of serial and parallel DNA data storage. c) The length and data of sentence DNA fragments. d) The NGS results of serial DNA data storage. e) A diagram of parallel processing in DCF. f–i) The gel electrophoresis results for PCR‐based random access of parallel DNA data storage (f), and the random‐access NGS results using primer files (g–i).

To process DNA data storage, the DNA droplet assembled using ligation in DCF has the benefits of programmable, serial, and parallel data assembly. The DNA word fragments were synthesized using a serial DNA data assembly to form the DNA sentence fragment (lane E of **Figure** **6**b) for “DNAdata Stores Apple Orange” (Figure 6a–c). The consensus NGS results of the DNA sentence data are shown in Figure 6d.

The serially synthesized DNA “DNAdata Store Parallelly” fragment was assembled with three different word data DNAs, “Apple,” “Orange,” and “Grape,” in a single droplet to split the synthesis for parallel DNA data storage processing (Figure 6e). The reverse address of the serially synthesized DNA fragment was assembled in parallel with three different splint DNAs, which were hybridized DNA word data of “Apple,” “Orange,” and “Grape” (Tables S2 and S4, Supporting Information). The DNA pool, assembled in series and parallel in DCF, had three different sentence DNA data fragments, “DNAdata Stores Parallelly Apple” (290 bps), “DNAdata Stores Parallelly Orange” (293 bps), and “DNAdata Stores Parallelly Grape” (209 bps) (lane F in Figure 6c). We used two strategies to read the multiple DNA data pools: (1) scanning the address sequences to access the data pool in silico and (2) using a primer‐based random‐access approach. In the in silico process, the NGS reads of the multiple DNA data pools were scanned and sorted using all address sequences to recognize file information such as size and position and the number of entries (Figure S11, Supporting Information). Three split DNA sequences were decoded in series and parallel to recover the digital data. PCR was performed on the DNA data pools using address primers to select the DNA files. The NGS results were then decoded to recover the digital data. Figure 6f–i presents the gel electrophoresis results, which show random access results with address primers through PCR and NGS. The programmable ordering assembly of DNA data in DCF can be conveniently manipulated to represent diverse DNA data, owing to the serial and parallel synthesis, due to the assembly address sequences. Moreover, the address sequences can be widely used as alignment markers in the decoding process.

### 2.6 Alignment using Address Sequences

In digital DNA data storage, primer sequences are pre‐defined information and can access DNA data in pools through PCR. Additionally, the primer address sequences, used for programmable data connections through splint ligation, can align with the DNA data file as a synchronization marker^[^ ^39^ ^]^ to improve the data fidelity and define the data in series and parallel. The data size and position can easily recover original digital data.

**Figure 7:**
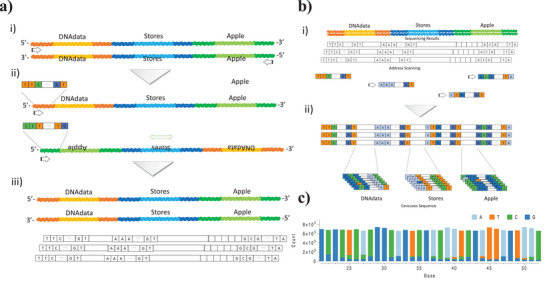
The decoding process for programmable droplet DNA data storage assembly. a) The decoding process with re‐alignment to the original information using the reverse primer information. b,c) Address sequence scanning (b), and the consensus sequencing of the DNA data (c).

**Figure** **7** shows the decoding protocol for digital DNA data synthesized through droplet DNA data assembly using multiple address information. The reverse data sequences in the synthesized DNA sentence, read by paired‐end sequencing, were searched using reverse primer information and flipped original sequence information. The flipped data provided approximately twice the coverage with high fidelity.^[^ ^44^ ^]^ Owing to the original ssDNA assembly with the splint DNA direction, the flipped DNA sentence data was serially converted from the 3′ to 5′ direction (Figure 7aii). The next step was to scan the address sequences (Figure 7b). After the DNA data size was assigned by scanning the last address sequence, the file information (positions and size of the data sequence) was searched and defined by scanning other address sequences. As the positions, data size, and order of DNA data were assigned by scanning the address sequences, the data sequences were statistically analyzed using consensus sequence and decoded original word data (Figure 7c), and the sentence data were represented (Figure 7bii). Moreover, the decoding protocol can search the parallel assembled DNA data by scanning multiple address sequences.

The address sequence assembly and searching method can efficiently optimize the data length and increase the sequence fidelity by aligning the address sequences. Moreover, decoding in de novo sequencing can be performed rapidly owing to assigning the data position and flipping the original sequence using reverse sequences. Address alignment is a suitable method for droplet DNA assembly to analyze serial and parallel assembled DNA data information.

### 2.7 Droplet Assembly of the DNA Data Pool

Due to synthetic lengths, DNA data storage molecules are stored in parallel in a DNA pool with index sequences. The DNA pools with common address sequences can be processed using droplet assembly in the DCF to manipulate the DNA pool files and create new information.

**Figure 8:**
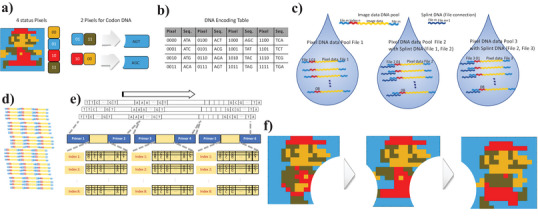
Programmable ordering assembly for DNA pools. a) The encoding process of four colors of pixel data. b) The two pixels one codon‐based encoding table. c) DNA pool with splint DNA in the droplet. d) The diagram of the assembled movie chip data DNA. e,f) The schematic diagram of the decoding process (e), and the movie clip images (Video S1, Supporting Information) (f).

**Figure** **8** shows the processing of parallel DNA data storage pools, in which a movie clip was created using three image files (0.27–0.28 kB) of 16 × 16 pixels with four colors. A pixel image file was encoded to DNA sequences through a two‐pixel per one codon code translation to prevent homopolymers, frequently repeated when the same pixel color is shown in a specific or background area.^[^ ^25^ ^]^ Three sequences were allocated based on two pixels (four bits of data) (Figure 8a). The DNA sequences produced by the two‐pixel‐based one codon code (1.3 bits per bps) did not create more than two repeated homopolymers (Figure S12, Supporting Information). The encoding protocol is provided in more detail in Supporting Information 13. The encoded pixel image file was stored in parallel in the synthesized 90 bps DNA pool with a two‐bps index and two 20 bps address sequences. Each DNA pool for the three “Super Mario” image files with splint DNAs in the droplet (Figure 8c) connected to create the movie clip file (12 kB). Figure 8e shows the parallel decoding process for aligning the data with the index sequences and constructing the image frame in each address sequence. After consensus sequencing, the DNA sequence reads were sorted using primers for the frames and by the index for the pixel order. The sequence data were decoded using binary information and arranged in 16 × 16 matrixes. Although the four‐color pixel images and movie clip files require pixel and color information for digital file conversion, the NGS result of the associated DNA pool was successfully decoded to produce the movie clip (Video S1, Supporting Information).

In our DCF system, three droplets containing 0.27–0.28 kB data in eight 90 bps oligos (33.7–35 B per oligo) created the movie clip file (12 kB) using linear droplet ligation. However, the droplet, which can contain 9.04 × 10^12^ copy oligos, has 303–315 GB storage capacity in the single droplet (× 1000 copies for ligation efficiency). The system could be expanded to logical droplet assembly, using logic circuit fluidics for DNA association^[^ ^11^ ^]^ instead of linear droplet ligation.

## 3 Discussion

Digital DNA data storage is a unique research focus on next‐generation information storage of materials because of its capacity for high‐density information. DNA molecules have been developed with limited functions, such as allowing random access using address primer information, but methods for processing DNA data are lacking. In this study, we demonstrate the programmable assembly of DNA data storage using address information in the DCF system. DNA sequences of 100 bps were assembled into five discrete size‐based DNA sequences using splint ligation to display the multi‐bit DNA information and programmable order assembly according to the splint DNA information. For DNA processing, DNA word data encoded in the sequence were combined in series and parallel, and single and multiple sentence data DNA pools to produce new information. The synthesized DNA pool contains sentence information accessed through PCR or decoded using the address primer information. During decoding, the data size and position in the sequences were defined in series and parallel to recover the original digital data using the address information easily. The sequences were aligned and sorted to improve fidelity. Finally, we demonstrate the programmable assembly of DNA pools that encode pixel images. The DNA pools of image frames assembled in series were sequenced and decoded in parallel to produce movie clips.

DNA storage is a valuable medium that can provide beneficial tools for high‐density ^[^ ^37^, ^45^ ^]^ and long‐term data storage^[^ ^46^, ^47^ ^]^ to replace traditional solid‐state storage materials such as compact disks, hard disk drives, and solid‐state drives. Furthermore, DNA, functioning as data‐bearing molecules in suspension, can be manipulated to process digital DNA data storage using programmable DNA assembly in the DCF system. Specifically, 303–315 GB droplets can provide a vesicle for discrete DNA data with programmable associations in the system. The synthetic ssDNA pool can allow a more reasonable data‐producing price and capacity.^[^ ^37^, ^48^ ^]^ Splint ligation enables sequence assembly, including synthesizing multiple DNA data pools. Including our data capacity, the system could be expanded to logical droplet assembly, using logic circuit fluidics for DNA association^[^ ^11^ ^]^ instead of linear droplet association. Even though we only demonstrated a few to tens kBs DNA data processing system using a 3 × 2‐inch droplet fluidics chip system with individual syringe pumps, the system can be extended to a scalable data processing machine with optimized processes using a room‐scale machine‐based system, such as a CatalogDNA machine^[^ ^49^ ^]^ and droplet fluidics system.^[^ ^28^, ^50^ ^]^

In addition, address sequence searching can provide high‐fidelity sequences and fast de novo sequence decoding and optimizes data density for DNA synthesis. We focused only on short‐sequence DNA for NGS. The processes in the droplet chip system are not fully automated and include NGS protocols, such as PCR and A‐tailing.^[^ ^51^ ^]^ However, the serial assembly of parallel DNA pools in droplets could be expanded for long‐sequence DNA data storage for Oxford nanopore sequencing, a real‐time sequencing technology.^[^ ^30^ ^]^ Furthermore, processing DNA using the droplet system can be extended to efficiently analyze large datasets in DNA data storage^[^ ^1^, ^52^ ^]^ and DNA computing.^[^ ^3^, ^17^, ^24^, ^53^ ^]^ Programmable DNA data assembly techniques in chips can potentially be used as valuable tools in memory computing and data storage for DNA through the enhanced development of DNA algorithms and discrete droplet systems.

## 4 Experimental Section

### The DCF System

The fluidic mold was designed by AutoCAD 2020 (Autodesk, Inc., San Francisco, CA, USA) and printed using Form 2 (Formlabs, Inc., Somerville, MA, USA) at the Device Lab (Incheon, Republic of Korea). The PDMS was cast in the mold to create the fluidics chip. The DCF system was constructed manually using NE‐1000 syringe pumps (New Era Pump Systems, Inc., Farmingdale, NY, USA) with a 1 ml syringe (BD, Inc., Franklin Lakes, NJ, USA). For the DNA droplet assembly process, DNA in the droplets of the main channel was mixed with T4 DNA ligase and ligase buffer (New England BioLabs, Inc., Notting Hill, Australia). The DNA molecular droplets were prepared by serially adding 2 µl of DNA data droplets with a 5 µl air gap in the capillary tube using a syringe pump. The droplet in the main channel was carefully moved and placed at the y‐junction of the channel. The injected droplet at the junction was associated with the droplet in the main channel. The associated droplet was then moved to the next y‐junction in the main channel to assemble the programmable discrete DNA droplet. The droplet assembly process was monitored using a Leica DMi8 inverted microscope (Leica Microsystems, Inc., Wetzlar, Germany). For DNA sequence ligation, the assembled droplets were incubated at room temperature for 20 min and moved to the next y‐junction. After assembly, the T4 DNA ligase enzyme was deactivated at 65 °C for 10 min to provide a consistent processing time and prevent unknown enzyme reactions during ligation and PCR.

### DNA Preparation

DNA sequences were designed using nupack.com and obtained from Integrated DNA Technologies, Inc. (Coralville, IA, USA) and Macrogen, Inc. (Seoul, Korea). All DNA sequences are listed in Supporting Information and DNA list file. For ligation, the 5′ ends of DNA sequences were phosphorylated with T4 polynucleotide kinase (New England BioLabs, Inc.) The phosphorylated DNA sequences were hybridized with splint DNA in the annealing process, during which the temperature was decreased from 95 to 30 °C by 1 °C min^−1^ in a SimpliAmp thermal cycler (ThermoFisher Scientific, Inc., Waltham, MA, USA). All DNA droplets were prepared to achieve a final concentration of 4 µm in 50 mm NaCl buffer (Samchun Pure Chemical Co., Seoul, Korea). In the 20 µl main droplet, the DNA samples were mixed with 400 U T4 DNA ligase (New England BioLabs, Inc., Notting Hill, Australia) in 1× reaction buffer (New England BioLabs, Inc., Notting Hill, Australia).

### The Encoding and Decoding Processes

The DNA data encoding and decoding processes were developed using Matlab 2021a (Mathworks, Inc., Natick, MA, USA). For the ASCII letter encoding of words, the text information could be represented as the ASCII code converted into binary information and stacked with RS ECC encoding for the seven bits error corrections. The binary information of the words was translated into the four DNA bases (A, T, C, and G). The encoded DNA letters were aligned with primer information for amplification and ligation. For two pixels, which were codon‐based encoding of the pixel images, 16 × 16‐pixel image frames, which have four color states per pixel, were decoded and linearly aligned. Each image frame was decoded using the same process. The four state colors were represented as 00, 01, 10, and 11. The binary information was translated by the codon DNA bases per two pixels. The encoded DNA sequences were fragmented with index sequences and aligned using primer information for DNA data oligo pools. The PIM DNA data, processed using NGS, were read and decoded by each decoding protocol. All DNA data and source codes are available in the GitHub (https://github.com/bioelectronicsbiooptics/Droplet‐DNA.git) and NCBI (PRJNA955202) databases.

### PCR and Detection

For conventional PCR, the samples with 300 nm of each primer (Integrated DNA Technologies, Inc., Coralville, IA, USA) were amplified in 0.2 mm dNTP (Ezynomics, Inc., Seoul, South Korea) and 1 U *Taq* polymerase (ThermoFisher Scientific, Inc., Waltham, MA, USA), using a SimpliAmp thermal cycler. For qPCR, the DNA samples were amplified with PowerUp SYBR Green Master Mix (Thermo Fisher Scientific, Inc., Waltham, MA, USA), using a QuantStudio 5 Real‐Time PCR System (Thermo Fisher Scientific, Inc., Waltham, MA, USA). The DNA and ligated DNA fragments were aliquoted to 1/40 000 to measure the fluorescent intensity using five standard samples diluted to 1/5 from 15.4 pg µl^−1^ to 24.2 fg µl^−1^. For gel electrophoresis, the DNA samples were visualized with 5 × SYBR green (Thermo Fisher Scientific, Inc., Waltham, MA, USA) and run on 1.5% (w/v) agarose gel (Sigma–Aldrich Company Ltd., St. Louis, MO, USA) in 1 × Tris/borate/EDTA buffer at 100 V for 40 min. The gels were imaged using a LED‐Transilluminator (Korea Ace Scientific Company Ltd., Seoul, South Korea) and an Amersham Typhoon 5 (Cytiva, Inc., Marlborough, MA, USA). Sanger sequencing and NGS were performed using an ABI PRISM 3730XL Analyzer and Illumina Miseq (Illumina, Inc., San Diego, CA, USA) from Macrogen, Inc.

### Statistical Analyses

For qPCR analysis, five standard samples (n = 3) of double‐stranded DNA were calculated using a DNA copy number calculator (https://www.thermofisher.com/). For the DNA quantity in the droplet, the copy numbers were multiplied by 40 000 aliquoted amounts.

## Conflict of Interest

Y.S. is the C.E.O. of Standard Bioelectronics. Co.

## Author Contributions

M.Y. and D.L. contributed equally to this work. M.Y. performed the experiment. D.L. analyzed and plotted data and revised the manuscript. J.K. encoded the data DNA sequences and decoded DNA sequencing files. Y.S. designed and supervised the experiment.

## Supporting information

Supporting Information

Supplemental Video 1

## Data Availability

The data that support the findings of this study are available from the corresponding author upon reasonable request.
