# Supplementary material for: Processing DNA Storage through Programmable Assembly in a Droplet‐Based Fluidics System
Source: Adv Sci (Weinh). 2023 Sep 26;10(32):2303197. doi: 10.1002/advs.202303197 (PMC10646262; doi:10.1002/advs.202303197)
Supplement: Supplementary file 1 — Supporting Information [file ADVS-10-2303197-s002.pdf]

ADVANCED SCIENCE
Open Access

Supporting Information

for *Adv. Sci.*, DOI 10.1002/advs.202303197

Processing DNA Storage through Programmable Assembly in a Droplet-Based Fluidics System

*Minsang Yu, Doyeon Lim, Jungwoo Kim and Youngjun Song**

Supporting Information

Processing DNA storage through programmable assembly in a droplet-based fluidics system

Minsang Yu[a], Doyeon Lim[a], Jungwoo Kim[a], and Youngjun Song[a,b*]

[a]Department of nano-bioengineering at Incheon National University, Academy-ro 119 Incheon, Korea, 22012

[b] StandardBioelectronics. Co., 511 Michuhol tower hall tower Gaetbeol-ro 12, Incheon, Korea, 21999

*Corresponding author: Y.Song (yjunsong@inu.ac.kr)

Supporting Information 1

For the macroscale fluidics mold of the PDMS fluidics chip, 3D printing was carried out to construct the complex structure with mm level height, due to the limitation of the sub-mm level height 2D pattern by photolithography process with a high viscosity photoresistant polymer, such as SU-8. Unlike our previous 3D printed microfluidics method, the 3D printed mold was not coated with a polymer to ensure a smooth surface. For the strong bonding, the

PDMS chip, which has a non-smooth surface, was bonded onto the PDMS substrate.

Supporting Information 2

To obtain droplets of a few microliters, a 3D mold was designed by AutoCAD 2022. The channels were designed to have 1 mm height and width at Figure S1. The 3D printed mold was practically cured to a ~ 2 mm channel level width, owing to UV intensity and polymer curing.

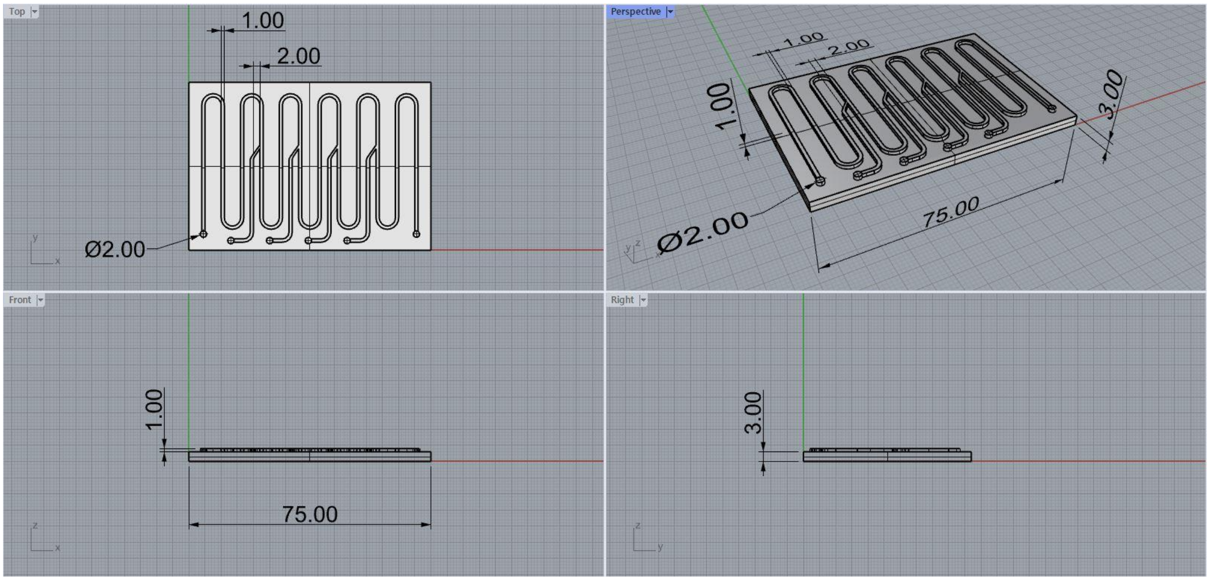

Figure S1. The 3D mold image of the droplet controlled fluidic system.

Supporting Information 3

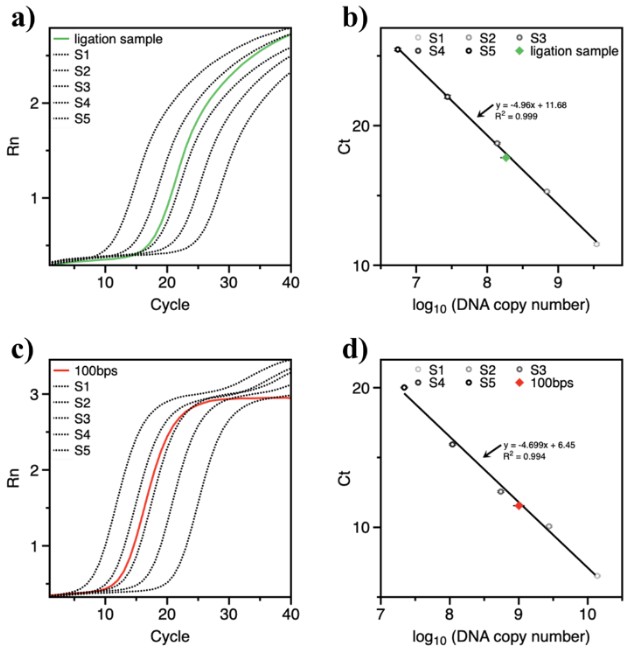

Figure S2. The quantitative PCR results. a) Amplification curve of 200 bps ligated sample. b) Standard curve of 200 bps ligated sample. c) Amplification curve of 100 bps sample. d) Standard curve of 100 bps sample.

Supporting Information 4

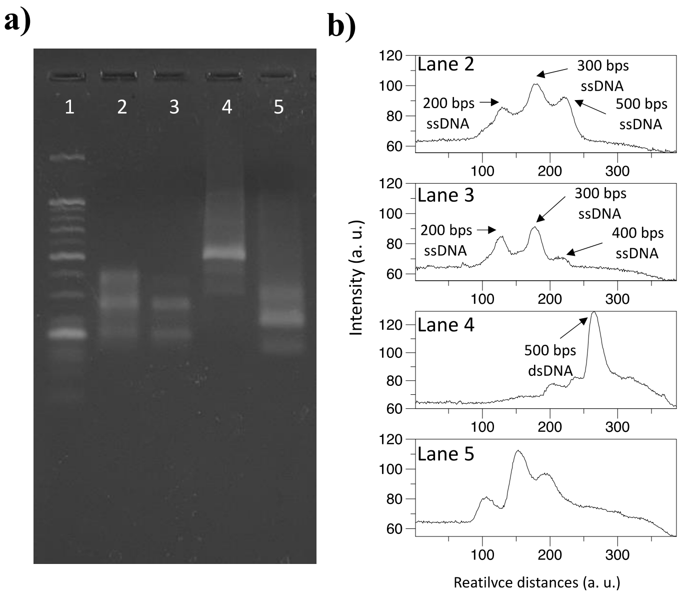

Figure S3. Splint DNA-ligated 500 bps fragment with and without ligation and PCR. a) The gel electrophoresis results of the splint DNA-ligated 500 bps fragment. b) Gel analysis of the splint DNA-ligated 500 bps fragment. (The ligated DNA in lane 2, the assembled DNA without ligation in lane 3, the ligated DNA with PCR in lane 4, and the assembled DNA with PCR in lane 5)

Supporting Information 5

Our 500 bps DNA samples were analyzed using Illumina Miseq, which can be read at 300 bps by paired-end reading. (Macrogen, Inc., Seoul, South Korea) For analysis of the reserved complementary sequences, the sequences were matched using the primer information. The sequencing results were aligned and statistically analyzed using MATLAB 2022a. Figure S2 and S3 show the consensus sequencing results with a high quality per base pair.

Table S1. Primer and splint DNA sequences for DNA A, B, C, D, and E.

DNA	Seq.
Primer 1 (DNA A)	Forward primer: CGT GCC AAC TGC ATT TAT GA<br>Reverse primer: TGT TGC CCG TCT CAC TGG TG
Primer 2 (DNA B)	Forward primer: CTG AAC AAG AAA AAT AAT ATC CCA TCC TAA TTT AC<br>Reverse primer: GTT TAA TAC CCG TTC TTG GAA TGA TAA GG
Primer 3 (DNA C)	Forward primer: TTT GAA TTA CCT TTT TTA ATG AGA ACA GTA CA<br>Reverse primer: CTA AGG GAA ACT ATT TAC AAA GCG AC
Primer 4 (DNA D)	Forward primer: AAC AGA TAA GTG CCG TCG AGA C<br>Reverse primer: TGG CGA GTC TCT GAG AGG T
Primer 5 (DNA E)	Forward primer: TCA TAG CGA TGC ACT AAC ACT TTT AC<br>Reverse primer: TCG CTC ATC TTA GGG TAA AAT CCG
Splint DNA 1 (DNA A-DNA B)	ATT TTT CTT GTT CAG TGT TGC CCG TCT CAC
Splint DNA 2 (DNA B-DNA C)	AAA AGG TAA TTC AAA GTT TAA TAC CCG TTC
Splint DNA 3 (DNA C-DNA D)	CGG CAC TTA TCT GTT CTA AGG GAA ACT ATT
Splint DNA 4 (DNA D-DNA E)	AGT GCA TCG CTA TGA TGG CGA GTC TCT GAG
Splint DNA 5 (DNA A-DNA D)	TGT TGC CCG TCT CAC CGG CAC TTA TCT GTT
Splint DNA 6 (DNA D-DNA C)	AAA AGG TAA TTC AAA TGG CGA GTC TCT GAG
Splint DNA 7 (DNA C-DNA B)	ATT TTT CTT GTT CAG CTA AGG GAA ACT ATT
Splint DNA 8 (DNA B-DNA E)	AGT GCA TCG CTA TGA GTT TAA TAC CCG TTC

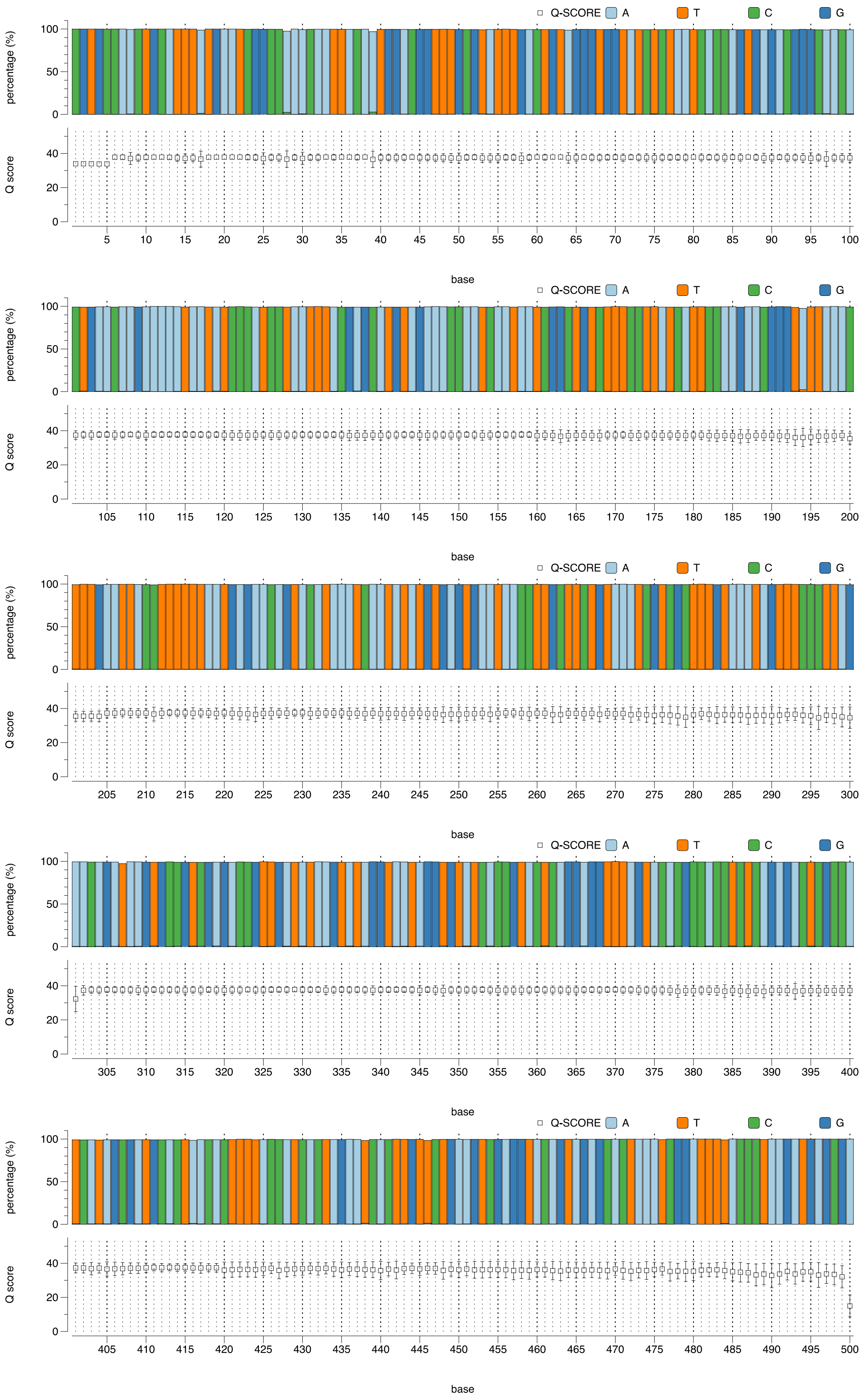

Figure S4. NGS results with the quality score for DNA fragments in the series order DNA A, B, C, D, and E.

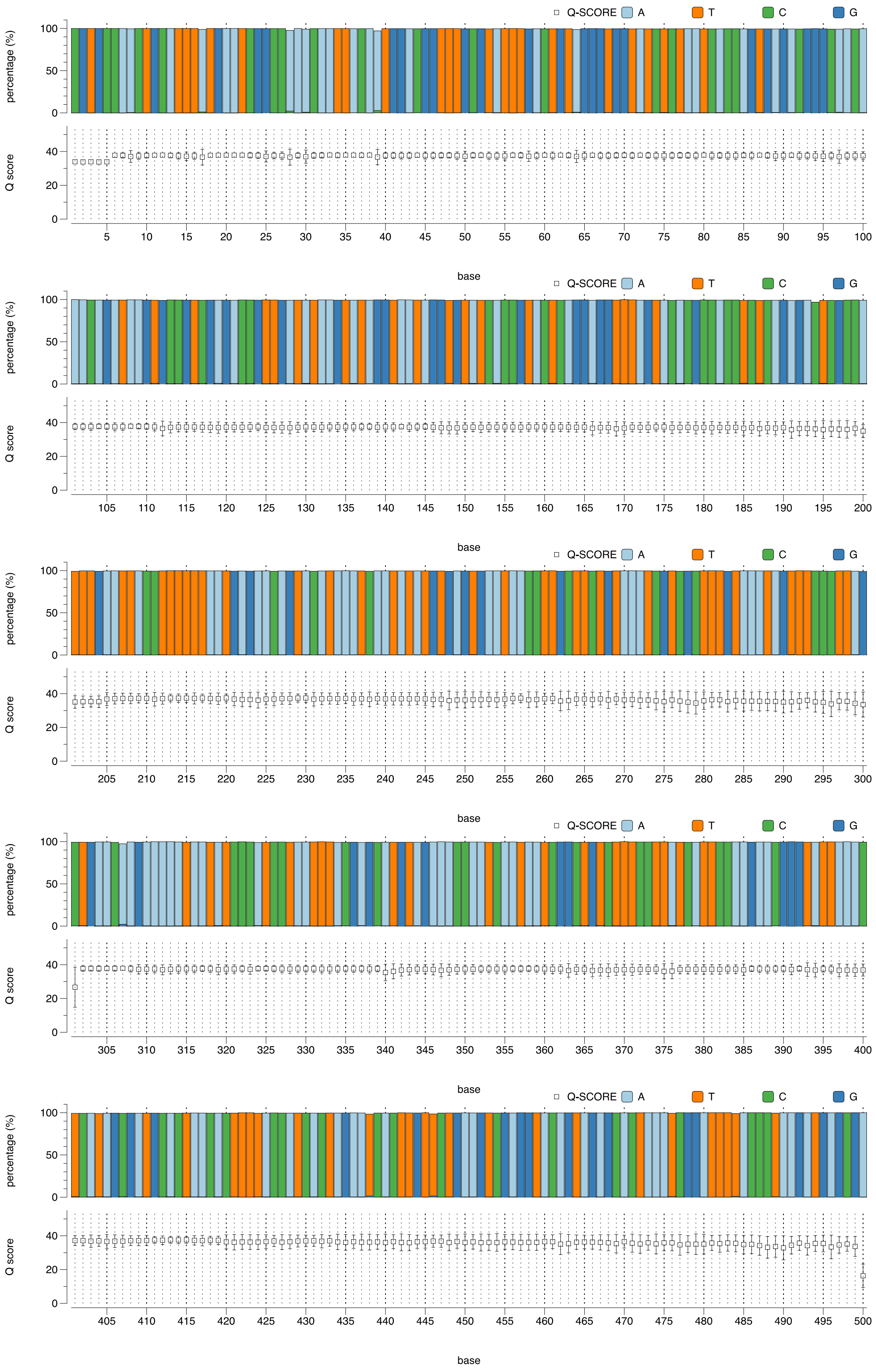

Figure S5. NGS results with the quality score for DNA fragments in the series order DNA A, D, C, B, and E.

Supporting Information 6

Table S2. The information of the Reed Solomon (RS) code and sequences of word DNA fragments.

	Data(word)	msg length	RS (n,k)	Galois Field	bp size	Forward Address	Payload with ECC	Backward Address
1	DNAdata	7	9,7	2^7	72	TTC GTT CGT CGT TGA TTG GT	GCG CTC ACG GCA GTC GAC AAT CGA TTA GTA AC	AAA CGG AGC CAT GAG TTT GT
2	Apple	5	7,5	2^7	65	CTG TCC ATA GCC TTG TTC GT	CGG CGA TGG TCA CTC CAT TAT TCG C	GCG GAA ACG TAG TGA AGG TA
3	Orange	6	8,6	2^7	68	TGT ATT TCC TTC GGT GCT CC	TTG CCA TCG ACT CTT CAT AGT TAC TCA C	TTT CGA CAA CGG TCT GGT TT
4	Grape	5	7,5	2^7	65	AGC CTT GTG TCC ATC AAT CC	TCG CCA TCG ACG ATC CAT TAG ATT G	TGC GCT ATG GTT TGG CTA AT
5	JamesWatson	11	13,11	2^7	86	GTC CAG GCA AAG ATC CAG TT	AAG TGG TCT ATA GTT GTT TAA CGA CAA TTG TTT CTA TAC CCC TGA C	ACC ACC GTT AGG CTA AAG TG
6	FrancisCrick	12	14,12	2^7	89	ATC CTG CAA ACG CAT TTC CT	ACG CCA TCG ACT CTT GAT GCT TGT TCG AAG TTG CTT GAT CCT CTC GGG A	ATG CCT TTC CGA AGT TTC CA
7	JohnVonNeumann	14	16,14	2^7	96	TAG CCT CCA GAA TGA AAC GG	AAG TTC TGA ACT CTA CCT TCT ATA CTC ACC ATA ATC TAT GGT ATA CTC TAG TGT TG	TTC AAG CCA AAC CGT GTG TA
8	Stores	6	8,6	2^7	66	TCC TCA GCC GAT GAA ATT CC	AAT TTG CAC TAG TGG TCC CCG GAC CT	TGT ACC ATC CGT TTG ACT GG
9	Has	3	5,3	2^7	56	GAA GAG TTT AGC CAC CTG GT	AAC AGA GAT TCC TGG T	AAG GCC AAT TCG CGG TTA TT
10	Likes	5	7,5	2^7	66	TAC CGC ATC CTT ATT CGA GC	AAA CCA GCA GCG AAT TCC CCG GAT AC	TCT GGT GCA AGC CAA TGA AA
11	Parallelly	10	12,10	2^7	87	CAA GAT TGT GGA CGA TTG GC	AAA AAT TAG TAG AAA AAC CGA ACT CGG AAT TCC CCC CCC CGT TAG TT	TGC AAT GTT TCC GTC GGT TT

Supporting Information 7

To create the single word DNA sequences with RS error correction code (ECC) redundancy, the codewords (the length of the codeword define n) were defined as the message words (the length of the message word was defined as k), which are based on 7 bits American Standard Code for Information Interchange (ASCII) code and two symbols, which were encoded with the RS ECC redundancy. For ECC encoding of the single word DNA fragments, the shortened RS (n, k) code[1], which has 2^7 Galois field applied on each word DNA sequence. n is code length and k is message length. In our protocol, the error can correct one alphanumeric character by 2 RS parities within 7 nt, which was located in end of the payload sequence. For suitable length parameters for k of the message, the word information was automatically implanted and encoded to DNA sequence by Matlab 2022a.

Additionally, for correction efficiency, the RS ECC codes, which can be corrected one character (ASCII 7 bits) and 7 bits, were compared between single sentence DNA in the sequencing file. The RS ECC code for one character was applied to the 'DNAdata' and 'Apple' sequence and the RS ECC code for 7 bits was applied to the 'Stores' sequence. Table S2 shows that the RS ECC bits correction as 200, while RS ECC ASCII character correction was 2 and 105.

Table S3. RS ECC code for correction of single.

	DNAdata (1 character)	Stores (7 bits)	Apple (1 character)
Reads of Fastq		889024	
Primer sorting (15 bps)	390304	646430	718336
Perfect match	353453	554590	613161
Error	36851	91840	105418
Error detection	36851	91839	105418
Error correction	36849	91639	105313
No correction	2	200	105

No detection	0	1	0

The decoding protocol for sorting and arranging word data in a sequence using primer indexing involves extracting the sentences from the payload section, determining the size and order of the sentences with a flexible length, and then arranging the sentences in a sequence using the primer. This ensures data integrity and minimizes errors, ultimately resulting in finalizing the sentence data through the process.

Supporting Information 8

Figure S5 displays the consensus sequencing outcome for DNA sentences (lane A, B, C, and D), which were constructed using DNA droplets. The primer sequences, indicated by diagonal lines, were excluded, and the payload data sequences containing 7nt RS parities were decoded in a sequential manner to form sentences.

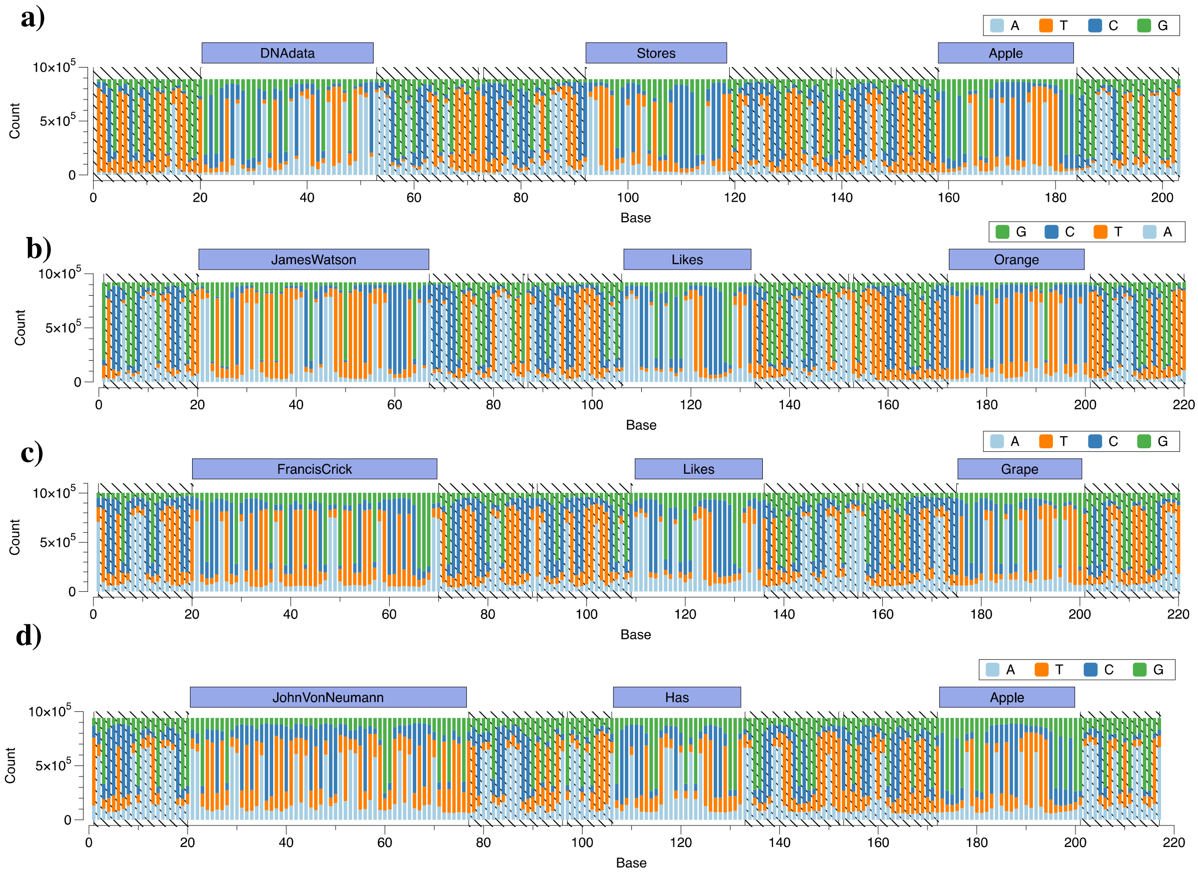

Figure S6. The sequencing results of sentence DNAs.

Supporting Information 9

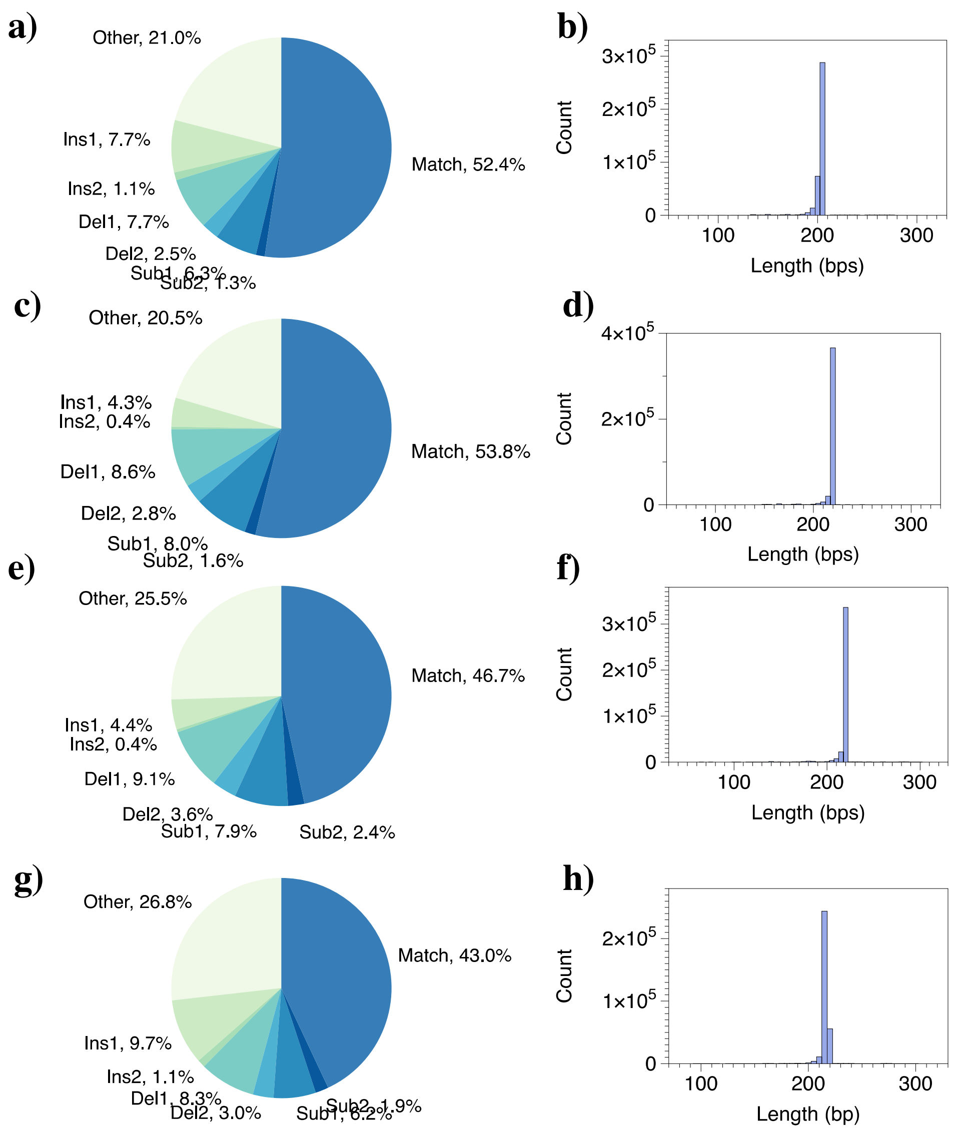

Figure S7. The sequencing analysis for sentence DNAs.

Supporting Information 10

Since the sentence DNAs were aligned using primer sequences, the consensus sequencing results and the count of perfect matching sequences were analyzed by the number of primer matched bases from 15 bps to 20 bps. Figure S5 shows the consensus sequencing results, including payload data with and without primer alignment.

As a result of the number of primer matched bases from 15 bps to 20 bps, we observed that the primer alignments count the number of sorted reads by the number of primer matched bases in 20 bps forward and reverse primers. Additionally, we monitored the perfect matched reads. Furthermore, the matched length of payload data was counted. To monitor the data DNA sequencing fidelity with ECC, we monitored the perfect matched reads, applying ECC.

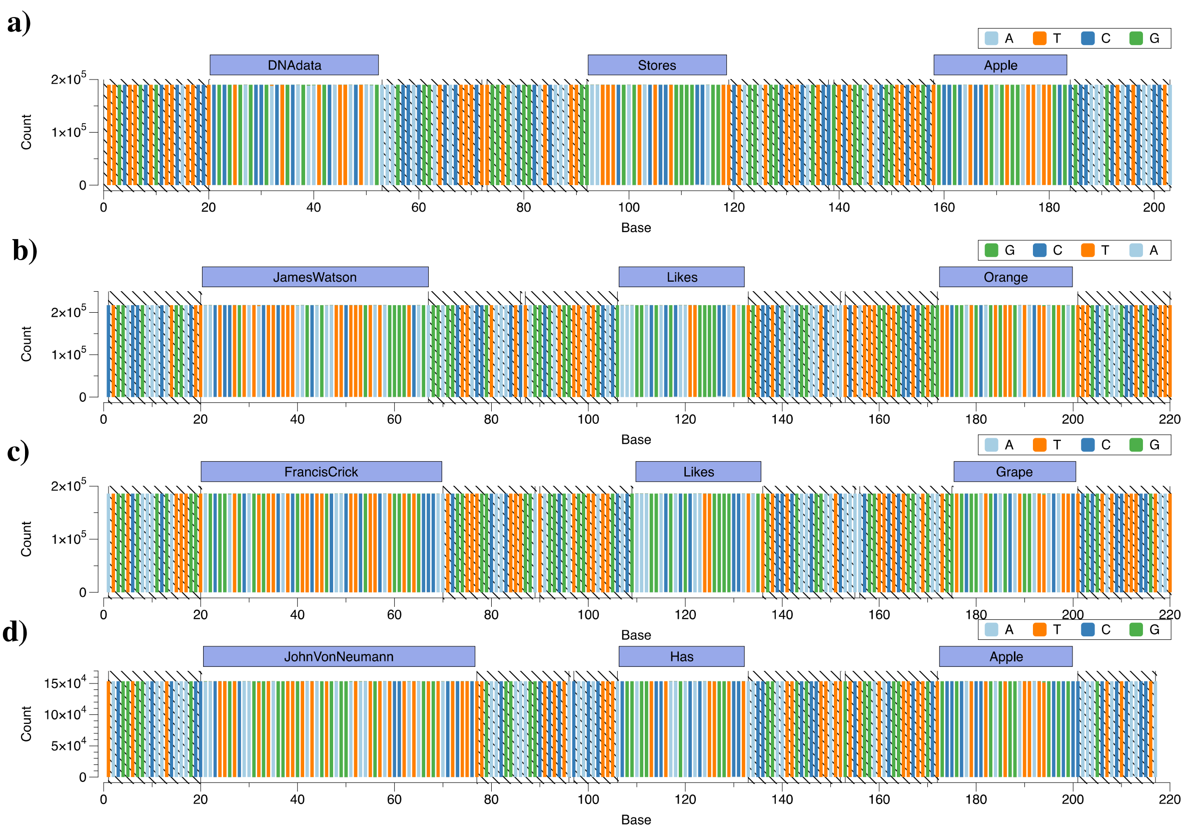

Figure S8. The sequencing results of sentence DNAs after primer sorting.

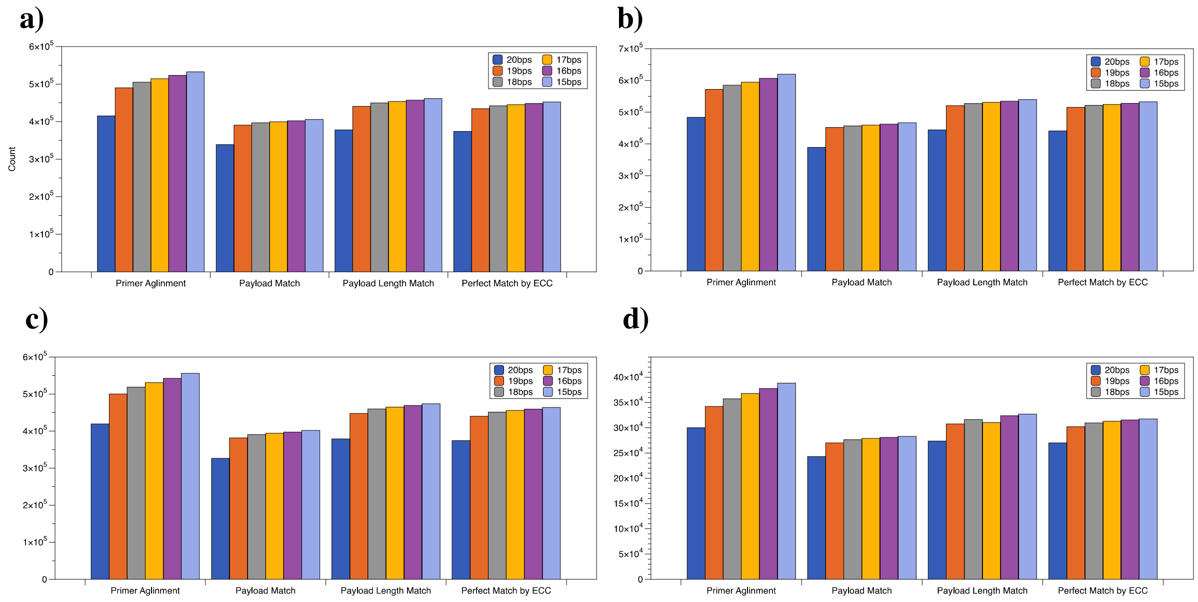

Figure S9. The number of counts for address sequence alignment, perfect match, payload data length match, and applying ECC by the matched address sequence, which was from 15 bps to 20 bps for sentence DNAs. (a) “DNAdata Stores Apple”, b) “JamesWatson Likes Orange”, c) “FrancisCrick Likes Grape”, and d) “JohnVonNeumann Has Apple”)

Supporting Information 11

Table S4. Splint DNA information and PCR primer information.

Sequences name	Oligonucleotide sequences (5’→3’)
Splint (1-8)	TTC ATC GGC TGA GGA ACA AAC TCA TGG CTC
Splint (8-2)	CAA GGC TAT GGA CAG CCA GTC AAA CGG ATG
Splint (2-3)	ACC GAA GGA AAT ACA TAC CTT CAC TAC GTT
Splint (5-10)	AAT AAG GAT GCG GTA CAC TTT AGC CTA ACG
Splint (10-3)	ACC GAA GGA AAT ACA TTT CAT TGG CTT GCA
Splint (6-10)	AAT AAG GAT GCG GTA TGG AAA CTT CGG AAA
Splint (10-4)	GAT GGA CAC AAG GCT TTT CAT TGG CTT GCA
Splint (7-9)	GTG GCT AAA CTC TTC TAC ACA CGG TTT GGC
Splint (9-2)	CAA GGC TAT GGA CAG AAT AAC CGC GAA TTG
Splint (8-11)	TCG TCC ACA ATC TTG CCA GTC AAA CGG ATG
Splint (11-2)	CAA GGC TAT GGA CAG AAA CCG ACG GAA ACA
Splint (11-3)	ACC GAA GGA AAT ACA AAA CCG ACG GAA ACA
Splint (11-4)	GAT GGA CAC AAG GCT AAA CCG ACG GAA ACA

Supporting Information 12

For random access of DNA storage, PCR based file selection is widely used. Since the DNA data were programmbly synthesized by primer information, we provided the random access process using software, which is searched by primer sorting. The DNA pools, which were synthesized with various information with primer sequences, were sequenced by NGS. The sequencing results file was aligned and sorted by primer information. Due to the base sequence position, the DNA data can be categorized as shown in Figure S7 c. Thus, the DNA data, which is associated by process in memory (PIM), has the merits for processing as well as decoding. Furthermore, the synthesized DNA data by PIM can allow random access by PCR technology, which is used to physically select DNA data files.

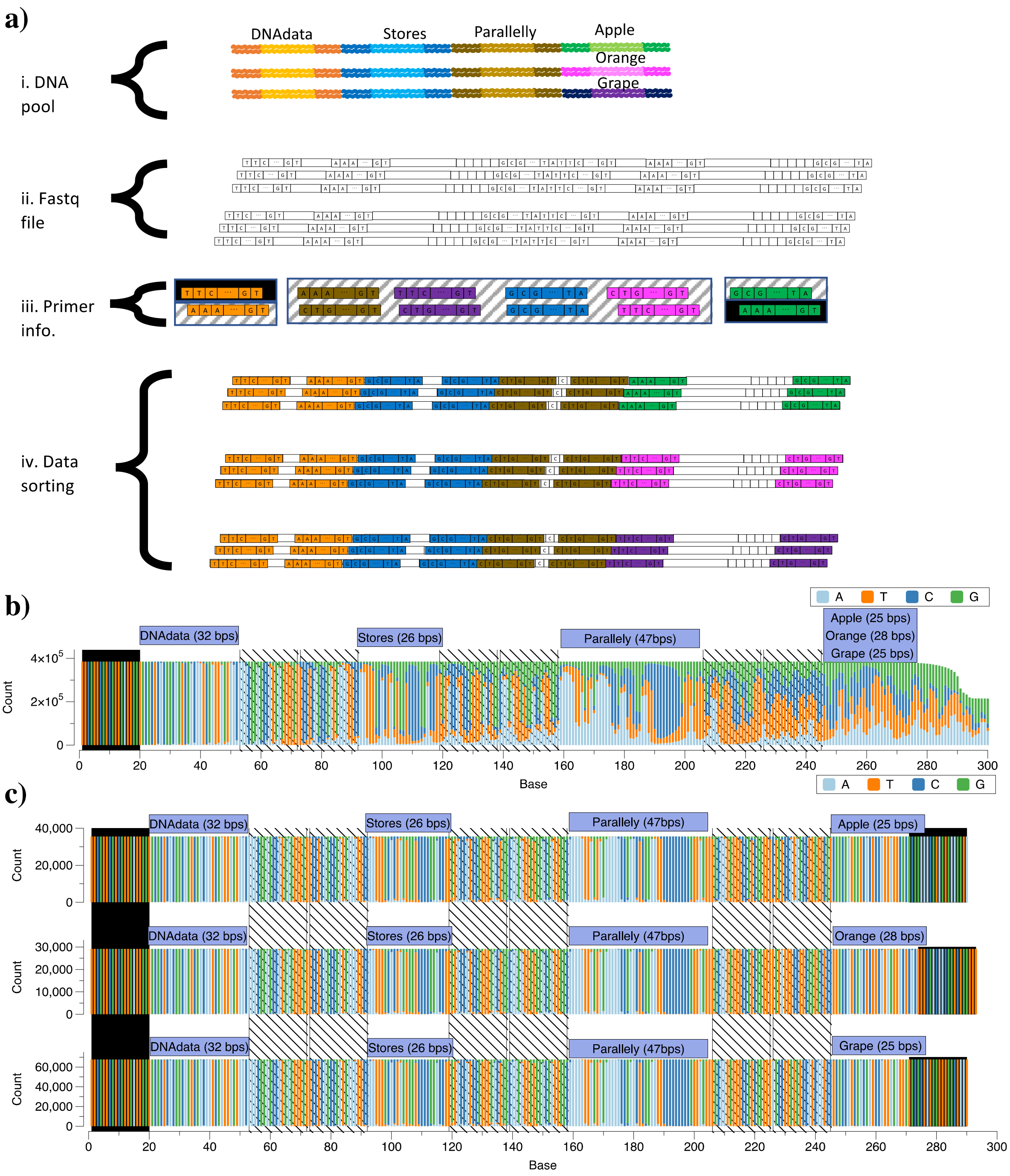

Figure S10. a) A diagram of the consensus sequencing for DNA pools by primer-based sorting. b) Consensus sequencing results of whole DNA pools. c) Consensus sequencing results of DNA pools by primer-based sorting.

Supporting Information 13

The two pixels codon code, which was encoded using 3 bps codons to represent the serial 2 pixels data (4 bits), has two main benefits. First, the repeated data can be represented as the sequence information without creating homopolymers, which is a critical error in sequence reading. Additionally, the codon information can not create more than two repeated homopolymers.

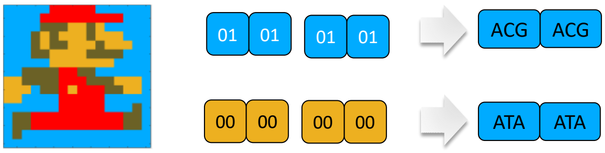

Figure S11. An example of the two pixels codon code.

Supporting Information 14

Table S5. The sequencing results of perfect matched sequence count sorted by primer matched sequence. (18 bps)

	Index 1	Index 2	Index 3	Index 4	Index 5	Index 6	Index 7	Index 8
Primer 1 /Primer 2	31685 (37991)	44927 (51760)	40309 (46155)	46442 (52868)	67847 (76262)	77317 (88749)	21203 (26260)	25100 (28898)
Primer 3 /Primer 4	23549 (28942)	28960 (33441)	33665 (38660)	25713 (29188)	28666 (31732)	23220 (26705)	38055 (44846)	12545 (14642)
Primer 5 /Primer 6	19140 (27174)	46778 (60865)	41864 (50076)	43955 (53737)	66130 (78555)	61017 (71699)	68072 (83666)	37820 (46797)

Supporting References

1. Yang, R., Chen, X. & Zhao, J. Shorten Reed-Solomon Code for Wireless USB. in *2014 Ninth International Conference on Broadband and Wireless Computing, Communication and Applications* 100–103 (2014). doi:10.1109/BWCCA.2014.57
